# Sensitivity analysis of selection bias: a graphical display by bias-correction index

**DOI:** 10.7717/peerj.16411

**Published:** 2023-11-16

**Authors:** Ping-Chen Chung, I-Feng Lin

**Affiliations:** 1 Department of Dentistry, Puzi Hospital, Ministry of Health and Welfare, Chiayi, Taiwan; 2 Institute of Public Health, School of Medicine, National Yang Ming Chao Tung University, Taipei, Taiwan

**Keywords:** Selection bias, Bias-correction, Sensitivity analysis, Health survey, Observational study

## Abstract

**Background:**

In observational studies, how the magnitude of potential selection bias in a sensitivity analysis can be quantified is rarely discussed. The purpose of this study was to develop a sensitivity analysis strategy by using the bias-correction index (BCI) approach for quantifying the influence and direction of selection bias.

**Methods:**

We used a BCI, a function of selection probabilities conditional on outcome and covariates, with different selection bias scenarios in a logistic regression setting. A bias-correction sensitivity plot was illustrated to analyze the associations between proctoscopy examination and sociodemographic variables obtained using the data from the Taiwan National Health Interview Survey (NHIS) and of a subset of individuals who consented to having their health insurance data further linked.

**Results:**

We included 15,247 people aged ≥20 years, and 87.74% of whom signed the informed consent. When the entire sample was considered, smokers were less likely to undergo proctoscopic examination (odds ratio (OR): 0.69, 95% CI [0.57–0.84]), than nonsmokers were. When the data of only the people who provided consent were considered, the OR was 0.76 (95% CI [0.62–0.94]). The bias-correction sensitivity plot indicated varying ORs under different degrees of selection bias.

**Conclusions:**

When data are only available in a subsample of a population, a bias-correction sensitivity plot can be used to easily visualize varying ORs under different selection bias scenarios. The similar strategy can be applied to models other than logistic regression if an appropriate BCI is derived.

## Introduction

In observational studies, estimates of the association between certain characteristics and outcomes of interest based on only a subsample of selected participants may be misleading because of selection bias. Selection bias occurs when certain characteristics influence the likelihood of inclusion or exclusion from a sample. This kind of bias cannot be corrected unless additional information regarding to those who were not selected to the sample is available. Statistical strategies for eliminating selection bias include standardization (Hernan & Robins, 2006), inverse probability weighting (Gustafson et al., 2013), and the Heckman two-stage sample selection model (Heckman, 1976). However, these methods require supplementary data to be obtained from the target population. When these data are not available, sensitivity analysis may be conducted to determine the possible magnitude and direction of the selection bias. However, this analysis requires user-specified selection bias parameters, which are based on subjective guesses or on other studies with high transportability (Infante-Rivard & Cusson, 2018).

The magnitude of selection bias on the coefficient of interest can be determined by the influence of outcome and covariates on their selection probability. Few studies, however, have analyzed how these selection probabilities are determined and incorporated as a means of quantifying the magnitude and direction of the potential selection bias in sensitivity analysis. In this study, we focused on practical issues regarding how to quantify and visualize the effect of selection bias in sensitivity analysis with regression models and demonstrated our strategy by using real data. A bias-correction index (BCI) was used to quantify potential selection bias through sensitivity analysis in the absence of external data. In the next sections, we begin with a brief theoretical introduction of six selection bias scenarios in a logistic regression setting (Hosmer & Lemeshow, 2000) and demonstrate how such bias can influence estimates of variable of interest. Finally, a bias-correction sensitivity plot was illustrated to present varying possible estimates under different selection bias scenarios by using data from the National Health Interview Survey (NHIS) in Taiwan, which was conducted among a subsample of individuals who consented to the national health insurance data linkage.

## Methods

### Selection bias due to of selected participants’ characteristics

Suppose that we are interested in the association between a binary exposure, X, and a binary outcome, Y, where Y =1 is the event of interest. Z denotes a potential confounder of the associations between X and Y. We assume the probability of interested outcome in a population given X and Z as logit  P(Y  = 1|X, Z) =  *β*_0_ + *β*_1_X + *β*_2_Z, where *β*_1_ is a parameter of interest. To describe how selection bias influences parameter estimation, we introduce the indicator S, which equals 1 when an individual is selected for the survey sample and 0 otherwise. The probability of an individual being willing to participate (or being selected) in a study can be expressed as a linear logistic form logit P(S = 1|Y , X, Z) =  *α*_0_ + *α*_1_Y  + *α*_2_X + *α*_3_Z. The linear logistic transformation of outcome prevalence with selection bias among selected participants can be written as $\text{logit}~\mathrm{P}(\mathrm{Y }=1{|}\mathrm{X},\mathrm{Z},~\mathrm{S}=1)=\varnothing \left( \alpha ;\mathrm{Y },\mathrm{X} \right) +{\beta }_{0}+{\beta }_{1}\mathrm{X}+{\beta }_{2}\mathrm{Z}.~\varnothing \left( \alpha ;\mathrm{Y },\mathrm{X} \right) $ is a function of coefficients from the equation of $\text{logit}\mathrm{P}(\mathrm{S}=1{|}\mathrm{Y },\mathrm{X}).~\varnothing \left( \alpha ;\mathrm{Y },\mathrm{X} \right) ~\text{is equal to}~{\alpha }_{1}+\log \frac{1+{\mathrm{e}}^{{\alpha }_{0}+{\alpha }_{2}\mathrm{X}+{\alpha }_{3}\mathrm{Z}}}{1+{\mathrm{e}}^{{\alpha }_{0}+{\alpha }_{1}+{\alpha }_{2}\mathrm{X}+{\alpha }_{3}\mathrm{Z}}} $, which is called BCI. As seen in the equation: logit P(Y  = 1|X, Z, S = 1), BCI is a term that corresponds to the difference between the estimate in the outcome model for the target and that from the sample. Ergo, the BCI represents the bias.

When Z is not only a confounder but also an effect measure modifier in the association between X and Y, it adds complexity to selection bias estimation. We also present another linear logistic form with an interaction term: logit  P(Y  = 1|X, Z, XZ) =  *β*_0_ + *β*_1_X + *β*_2_Z + *β*_3_XZ, where *β*_1_ is a parameter of interest. Z denotes effect measure modifier. The probability of an individual being willing to participate in a study is written as logit P(S = 1|Y , X, Z, XZ) = *α*_0_ + *α*_1_Y  + *α*_2_X + *α*_3_Z + *α*_4_XZ. The outcome prevalence model with selection bias among the selected participants can be written as $\text{logit}~\mathrm{P}(\mathrm{Y }=1{|}\mathrm{X},\mathrm{Z},\mathrm{XZ},~\mathrm{S}=1)=~\varnothing \left( \alpha ;\mathrm{Y },\mathrm{X} \right) +{\beta }_{0}+{\beta }_{1}\mathrm{X}+{\beta }_{2}\mathrm{Z}+{\beta }_{3}\mathrm{XZ}.\varnothing (\alpha ;\mathrm{Y },\mathrm{X})$ is equal to ${\alpha }_{1}+\log \frac{1+{\mathrm{e}}^{{\alpha }_{0}+{\alpha }_{2}\mathrm{X}+{\alpha }_{3}\mathrm{Z}+{\alpha }_{4}\mathrm{XZ}}}{1+{\mathrm{e}}^{{\alpha }_{0}+{\alpha }_{1}+{\alpha }_{2}\mathrm{X}+{\alpha }_{3}\mathrm{Z}+{\alpha }_{4}\mathrm{XZ}}} $.

There may be many factors influencing participants’ willingness to participate in a study. ∅(*α*; Y , X) can take different forms, depending on selection probabilities, and these differences directly affect the outcome model. Six scenarios of participant selection models are listed below. Scenarios 1-5 are based on the outcome model without interaction in the population, and Scenario 6 represents a general form of BCI with selection probability depending on Y, X, Z, and XZ.

• Scenario 1: The selection of participants is affected by neither Y nor X.

Selection model: logit P(S = 1|Y , X, Z) = P(S = 1) = *α*_0_

Outcome model with selection bias among selected participants: logit P(Y  = 1|X, Z, S = 1) = *β*_0_ + *β*_1_X + *β*_2_Z

• Scenario 2: The selection of participants is affected only by Y.

Selection model: logit P(S = 1|Y , X, Z) = P(S = 1|Y ) = *α*_0_ + *α*_1_Y

Outcome model with selection bias among selected participant: logit P(Y  = 1|X, Z, S = 1) = ${\beta }_{0}~+{\beta }_{1}\mathrm{X}+{\beta }_{2}\mathrm{Z}+ \left. \left( {\alpha }_{1}+\log \frac{1+{\mathrm{e}}^{{\alpha }_{0}}}{1+{\mathrm{e}}^{{\alpha }_{0}+{\alpha }_{1}}} \right. \right) $

• Scenario 3: The selection of participants is affected only by X.

Selection model: logit P(S = 1|Y , X, Z) = P(S = 1|X) = *α*_0_ + *α*_2_X

Outcome model with selection bias among selected participant: logit P(Y  = 1|X, Z, S = 1) = *β*_0_ + *β*_1_X + *β*_2_Z

• Scenario 4: The selection of participants is affected by both Y and X.

Selection model: logit P(S = 1|Y , X, Z) = P(S = 1|Y , X) = *α*_0_ + *α*_1_Y  + *α*_2_X

Outcome model with selection bias among selected participant: $\text{logit}\mathrm{P}(\mathrm{Y }=1{|}\mathrm{X},\mathrm{Z},\mathrm{S}=1)=~{\beta }_{0}~+{\beta }_{1}\mathrm{X}+{\beta }_{2}\mathrm{Z}+~ \left. \left( {\alpha }_{1}+\log \frac{1+{\mathrm{e}}^{{\alpha }_{0}+{\alpha }_{2}\mathrm{X}}}{1+{\mathrm{e}}^{{\alpha }_{0}+{\alpha }_{1}+{\alpha }_{2}\mathrm{X}}} \right. \right) $

• Scenario 5:The selection of participants is affected by Y, X and Z.

Z is a confounder variable.

Selection model: P(S = 1|Y , X, Z) = P(S = 1|Y , X, Z)  = *α*_0_ + *α*_1_Y  + *α*_2_X + *α*_3_Z

Outcome model with selection bias among selected participant: $\text{logit}\mathrm{P} \left( \mathrm{Y }=1{|}\mathrm{X},\mathrm{Z},\mathrm{S}=1 \right) ={\beta }_{0}~+{\beta }_{1}\mathrm{X}+{\beta }_{2}\mathrm{Z}+~ \left. \left( {\alpha }_{1}+\log \frac{1+{\mathrm{e}}^{{\alpha }_{0}+{\alpha }_{2}\mathrm{X}+{\alpha }_{3}\mathrm{Z}}}{1+{\mathrm{e}}^{{\alpha }_{0}+{\alpha }_{1}+{\alpha }_{2}\mathrm{X}+{\alpha }_{3}\mathrm{Z}}} \right. \right) $

• Scenario 6:The selection of participants is affected by Y, X and Z.

Z is an effect measure modifier.

Selection model: P (S = 1 | Y,X,Z,XZ) = P(S = 1 | Y, X,Z,XZ) = *α*_0_ + *α*_1_Y  + *α*_2_X + *α*_3_Z + *α*_4_XZ

Outcome model with selection bias among selected subjects: $\text{logit}\mathrm{P} \left( \mathrm{Y }=1{|}\mathrm{X},\mathrm{Z},\mathrm{XZ},\mathrm{S}=1 \right) =~{\beta }_{0}~+{\beta }_{1}\mathrm{X}+{\beta }_{2}\mathrm{Z}+{\beta }_{3}\mathrm{XZ}+ \left. \left( {\alpha }_{1}+\log \frac{1+{\mathrm{e}}^{{\alpha }_{0}+{\alpha }_{2}\mathrm{X}+{\alpha }_{3}\mathrm{Z}+{\alpha }_{4}\mathrm{XZ}}}{1+{\mathrm{e}}^{{\alpha }_{0}+{\alpha }_{1}+{\alpha }_{2}\mathrm{X}+{\alpha }_{3}\mathrm{Z}+{\alpha }_{4}\mathrm{XZ}}} \right. \right) $

A detailed derivation is shown in Appendix S1. In Appendix S1, it is shown that the probability P (Y = 1 | X, Z, *S* = 1) can be expressed as a function of P (Y | X, Z) and P (S | Y, X, Z). This observation makes it possible to express P (Y = 1 | X, Z, *S* = 1) as an equation containing both ∅(*α*;Y,X) and *β*_0_ + *β*_1_X + *β*_2_Z.

### Correction for selection bias and sensitivity analysis

Scenarios 4–6 indicate that when selection probability is influenced by both X and Y, the coefficient of X (the parameter of interest) is biased. For the outcome model without interactions between X and Z (Scenario 5), where selection probability is affected by X, Y, and Z, a BCI is as follows. Bias correction index (BCI) = ${\alpha }_{1}+\log \frac{1+{\mathrm{e}}^{{\alpha }_{0}+{\alpha }_{2}\mathrm{X}+{\alpha }_{3}\mathrm{Z}}}{1+{\mathrm{e}}^{{\alpha }_{0}+{\alpha }_{1}+{\alpha }_{2}\mathrm{X}+{\alpha }_{3}\mathrm{Z}}} $. When external data are available to estimate the BCI, the selection bias may be corrected by including the BCI as an offset in the outcome model. However, when external data are not available, the conditional probability of selection P (S = 1 | Y, X, Z) with the selection parameters (*α*_0_,  *α*_1_,  *α*_2_,  *α*_3_) is unknown. To quantify the magnitude of bias for the odds ratio (OR) of interest, selection parameters (*α*) can be determined based on the researchers’ understanding of the participants. Alternatively, *α* can be set within a range of values. We performed sensitivity analyses by assuming selection probability model with different parameters (*α*_1_, *α*_2_) and presents the results using a graphical display. In the next section, we will illustrate how to construct a bias-correction sensitivity plot, which shows varying possible bias-corrected ORs under different magnitudes of selection parameters.

### Application: analysis of survey data with potential selection bias

### National Health Interview Survey (NHIS)

The NHIS in Taiwan involves a national representative sample and has been conducted every four years since 2001. The survey is conducted using a multistage stratified systematic sampling design, with sample selection based on probability proportional to size. Goodness-of-fit tests revealed no significant differences of urbanization level, sex, and age between the survey sample and household registration data (National Health Research Institutes & Health Promotion Administration (HPA) of the Ministry of Health and Welfare (MOHW) of Taiwan, 2019).

The NHIS database can be linked to Taiwan’s National Health Insurance Research database (NHIRD) to explore the associations between disease, health utilization outcomes, and health behaviors. The National Health Insurance is a mandatory program for all citizens in Taiwan with coverage rate over 99% (Hsieh et al., 2019). For NHIS data to be linked with the NHIRD, participants in the NHIS must provide written informed consent. The following section will illustrate the practice of a sensitivity analysis using the NHIS data of only those who consented to the data linkage.

### The NHIS questionnaires and variables

We used the 2001 NHIS database to perform the sensitivity analysis because these data are often used to link to the NHIRD or cancer registries for long-term follow-up studies (Dai et al., 2020; Lo et al., 2022). This study was approved by the Institutional Review Board (IRB) of National Yang-Ming University (YM108041E).

Interviewers asked all participants through standard questionnaires and collected basic demographic data including age, sex, marital status and education level. Because smokers have a higher risk of colitis and colorectal cancer (Giovannucci, 2002; Srivastava et al., 1990), we hypothesized that nonsmokers have greater health consciousness and are more willing to undergo proctoscopic examination, which served as an example to introduce BCI. Participants were asked whether they had ever smoked in their lives (binary; X; 1 = yes, 0 = no), had undergone a proctoscopic examination in the past year (Y; 1 = yes, 0 = no), and had signed the informed consent for NHIRD linkage (S; 1 = yes, 0 = no).

The parameter of interest was the OR of the association between proctoscopic examination (Y) and smoking status (SMOKE) after adjusting for sex. We performed a sensitivity analysis assuming that data were only available for participants who consented to NHIRD linkage (*S* = 1). Details regarding variable information and model specifications are described in the following sections.

The main model can be presented as follows: logit P (Y = 1 | smoke, sex) = *β*_0_ + *β*_1_smoke + *β*_2_sex.

Based on previous literature (Tolonen, Dobson & Kulathinal, 2005), health behavior may influence the participants’ willingness to participate in a study. We supposed that both proctoscopic examination (Y) and smoking status (SMOKE) were related to study participation (*S* = 1) and that sex was not. The selection model can be presented as follows: $\text{logit}\mathrm{P} \left( \mathrm{S}=1{|}\mathrm{Y },\text{smoke},\mathrm{sex} \right) ={\alpha }_{0}+{\alpha }_{1}\mathrm{y}+{\alpha }_{2}\text{smoke}+{\alpha }_{3}\mathrm{sex},\text{where}~{\alpha }_{3}=0$.

The bias-corrected model is rewritten as follows (Scenario 4): $\text{logit}\mathrm{P}(\mathrm{Y }=1{|}\text{smoke},\mathrm{sex},\mathrm{S}=1)={\beta }_{0}+ \left. \left( \widehat{{\alpha }_{1}}+\log \frac{1+{\mathrm{e}}^{\widehat{{\alpha }_{0}}+\widehat{{\alpha }_{2}}\text{smoke}}}{1+{\mathrm{e}}^{\widehat{{\alpha }_{0}}+\widehat{{\alpha }_{1}}+\widehat{{\alpha }_{2}}\text{smoke}}} \right. \right) +{\beta }_{1}\text{smoke}+{\beta }_{2}\mathrm{sex}$, where BCI = $\widehat{{\alpha }_{1}}+\log \frac{1+{\mathrm{e}}^{\widehat{{\alpha }_{0}}+\widehat{{\alpha }_{2}}\text{smoke}}}{1+{\mathrm{e}}^{\widehat{{\alpha }_{0}}+\widehat{{\alpha }_{1}}+\widehat{{\alpha }_{2}}\text{smoke}}} $ .

In practice, researchers may determine the range of *α* based on their prior understanding of the relationship between S and other variables. A range of positive and negative *α* values are included if no prior information is available. The value of *α*_0_ is set as 1.96 on the basis of the data, which indicates that marginal proportion of individuals that consented to linkage P(*S* = 1) was approximately 88%. The bias parameters (*α*_1_, *α*_2_) were set to between −2.5 and 2.5, with an increment of 0.3 for each value. The BCIs were calculated using varying combinations of (*α*_1_, *α*_2_). Then, the bias-corrected ORs of SMOKE and Y were calculated using different BCIs. A bias-correction sensitivity plot was performed by BCI to understand how selection bias influenced the estimate of the variable of interest (exposure-outcome relationship).

In addition to the sensitivity analysis, we also calculated the OR without selection bias (*i.e.,* the OR based on the entire data) for demonstration, so that readers can observe the influence of varying BCIs. In practice, the OR without selection bias is unknown. However, the data of individuals who did not provide informed consent for NHIRD linkage (*S* = 0) are available. Therefore, in the present study, we were able to demonstrate the difference between the target estimate based on the entire data obtained from the main model and that based on the selected sample obtained from the outcome model in the bias-correction sensitivity plot.

All analyses were conducted two-sided with alpha set at 0.05. We conducted all statistical analyses and generated all charts by using lme4 (Bates et al., 2015), jtools (Long, 2020) and ggplot2 (Wickham, 2016) packages of R (version 4.0.0; R Core Team, 2020).

## Results

**Figure 1:**
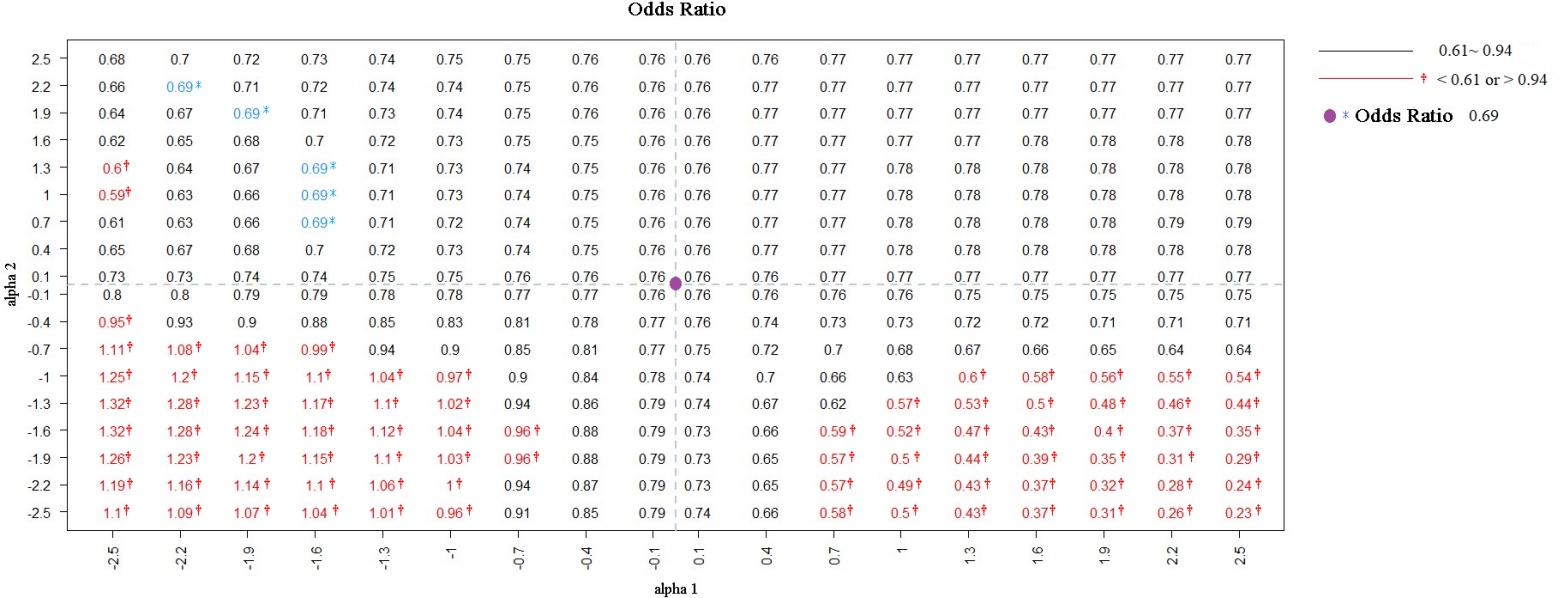
ORs were adjusted by BCI in diûerent degrees of selection bias from NHIS selected sample. Note. Purple dot is the intersection of the two dotted lines representing the OR = 0.76 among the selected sample (S = 1). The OR = 0.69 was marked in blue (unobserved).

**Figure 2:**
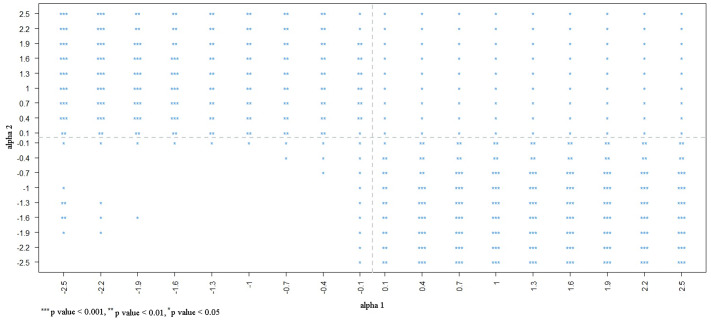
Statistical significance in different degrees of selection bias from NHIS selected sample.

We identified 15,247 individuals aged ≥20 years from the 2001 NHIS, 87.74% of whom provided informed consent for their data to be linked. Among the consenting population, smokers were less likely to undergo proctoscopic examination than nonsmokers were (OR: 0.76; 95% CI [0.62–0.94]). To evaluate the potential influence of selection bias, a sensitivity analysis was performed. Figure 1 presents the estimates corrected by BCI under different degrees of selection bias. The estimated OR based on the selected sample (*S* = 1) is marked in purple. In the bias-correction sensitivity plot, the bias-corrected parameters [log(OR)] beyond or below two standard errors (0.21) of the estimate obtained from the selected sample (OR = 0.76) corresponded to bias parameters *α*_1_ ≤−1.0 and *α*_2_ ≤ −1.0, and *α*_1_ ≥ 1.0 and *α*_2_ ≤ −1.3, which are marked in red. When the bias parameters (*α*1, *α*2) were extremely negative (≤−1.0), the smoking effect changed from being a risk factor to a protective factor for proctoscopic examination, rendering the significant association nonsignificant. At values < −0.4, the significant association between smoking status and proctoscopic examination became nonsignificant (Fig. 2).

For illustrative purpose, if we performed the same analysis using the entire data without selection bias, the estimated OR was 0.69 (95% CI [0.57–0.84]; in Fig. 1), showing that smokers were less likely to undergo proctoscopic examination, which is similar to the results obtained using only the selected sample (*S* = 1). However, the OR of 0.69 was not observed in practice when only the data of a selected sample were available. If we wanted to get an OR of 0.69 based on the selected sample, we would need to set *α*_1_ at approximately −1.6 to −2.2 and *α*_2_ at about 0.7 to 2.2. This indicated that individuals who underwent proctoscopy and smokers were 0.1–0.2 and 2–9 times, respectively, more likely to sign an informed consent form. When the bias parameter *α*_1_ took an extreme value and *α*_2_ took extreme negative values (far away from 0), the bias-corrected OR was markedly different from had large difference with the estimate based on the entire data without selection bias.

## Discussion

In our analysis of the data from individuals who consented to NHIRD linkage, the estimated OR (0.76) was slightly higher than that (0.69) based on the entire data set, implying minor selection bias.

When *α*_1_ and *α*_2_ were both less than −1 (*i.e.,* smokers had odds of participation of 0.37 [exp(−1)] or even lower than that of nonsmokers), the negative association between smoking status and proctoscopic examination had the potential to become a positive association (*lower left red area* of Fig. 1). When *α*_1_ was greater than 1.9 (*i.e.,* those who underwent proctoscopic examination tended to participate in the study) and *α*_2_ was less than −1.9 (*i.e.,* smokers had odds of participation of 0.15 [exp(−1.9)] or even lower than that of nonsmokers), the negative association between smoking status and proctoscopic examination had the potential to be exaggerated (*lower right red area* of Fig. 1). However, given that these two extreme situations are unlikely in practice, we can consider the estimates from the selected sample to be reliable.

Our results reveal the following scenarios. When selection probability is associated with neither the main predictor (X) nor the outcome (Y) (Scenario 1), the OR point estimate is unbiased. However, because the sample size of the selected sample is smaller than the entire sample, the confidence interval is wider. When selection probability is only associated with the main predictor (X) and not with the outcome (Y) (Scenario 3), the OR point estimate is unbiased. When selection probability is only associated with the outcome (Y), not with the main predictor (X) (Scenario 2), the OR point estimate is unbiased but the intercept changes. When selection probability is associated with both the main predictor (X) and outcome (Y) (Scenario 4), the OR point estimate and intercept are both biased.

Thompson & Arah (2014) used inverse probability weighting and externally obtained bias parameters to adjust selection bias, and this method can be applied to any type of observational study. The Heckman model includes a sample selection equation and main equation, and the main equation links the covariates of interest to the outcome (Heckman, 1976). However, implementing these methods can be challenging when external data containing information regarding the characteristics of the underlying source population are not available.

Through a bias-correction sensitivity plot, we can easily figure out the possible range of estimates obtained from a selected sample. The worst scenario with respect to selection bias occurs when participant selection in a specific study is related to both the exposure and outcome of the study. However, by evaluating the association between selection probability and key variables, researchers can quantify the magnitude of bias and boost confidence in the estimate of interested exposure-outcome relation, even when the data of non-participants are unavailable.

The BCI in this study was derived using a logistic outcome model, and a similar strategy may be applied for different outcome models. For example, when the main outcome model is a linear regression with a continuous outcome variable (Y), because the association of the expected value of Y is linear to the coefficients, a BCI can be constructed by the selection model.

This study has some limitations. In practice, the bias parameters in developed selection model need to be determined before the model is implemented, and justification for selected values is quite arbitrary. Researchers can select a broad but reasonable range of parameter values to figure out possible outcomes. However, interpretation of the association would not be easier because of the broad range of values.

## Conclusions

This study presents a visualized sensitivity analysis by given reasonable selection parameters to obtain the magnitude and direction of selection bias. We demonstrated the application of this analysis by using real-world data and provided a step-by-step explanation to guide readers of its implementation. When selection bias is unavoidable, we encourage researchers to be cautious and perform bias-correction sensitivity plot by following our steps, especially when no external data are available for correction. Our strategy can be applied to models other than logistic regression if an appropriate BCI is derived.

## Supplemental Information

10.7717/peerj.16411/supp-1 Appendix S1 Appendix

## Additional Information and Declarations

### Data Availability

The following information was supplied regarding data availability: The raw data are managed by the Health and Welfare Data Center (HWDC, https://dep.mohw.gov.tw/DOS/cp-5119-59201-113.html), Ministry of Health and Welfare of Taiwan. Access to the raw data requires a project-base application to the Taiwan government. The application is available at https://dep.mohw.gov.tw/DOS/lp-5146-113.html.
