# Supplementary material for: Sensitivity analysis of selection bias: a graphical display by bias-correction index
Source: PeerJ. 2023 Nov 16;11:e16411. doi: 10.7717/peerj.16411 (PMC10657564; doi:10.7717/peerj.16411)
Supplement: Appendix S1 [file peerj-11-16411-s001.docx]

Appendix. Inference of estimator correction in different selection bias scenarios

1. Scenario 1：Suppose the selection of participants is not affected by Y, X and Z.

Selection model: $P(S=1|Y,X,Z)=P(S=1)$

Outcome model: $logit P\left( Y=1 \right|X,Z, S=1)=log\frac{P(Y=1|X,Z)}{P(Y=0|X,Z)}= \beta_{0}+\beta_{1}X+\beta_{2}Z$


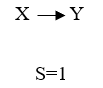


Scenario 1 A DAG representing marginally dependent X and Y without additional conditional (on S = 1) dependency.

DAG: directed acyclic graph.

X is the exposure; Y is the outcome; S is an indicator of sample selection.

(2) Scenario 2：Suppose the selection of participants is only affected by Y.

Selection model: $P(S=1|Y,X,Z)=P(S=1|Y)$

Outcome model: $logit P\left( Y=1 \right|X,Z, S=1)=log\frac{P(Y=1|X,Z)P(S=1|Y=1)}{P(Y=0|X,Z)P(S=1|Y=0)}= \beta_{0}{+ \beta}_{1}X+\beta_{2}Z+(\alpha_{1}+log\frac{1+e^{\alpha_{0}}}{1+e^{\alpha_{0+}\alpha_{1}}})$


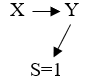


Scenario 2 A DAG representing marginally dependent X and Y, and conditionally (on S = 1) dependent Y.

(3) Scenario 3：Suppose the selection of participants is only affected by X.

Selection model: $P(S=1|Y,X,Z)= P(S=1| X)$

Outcome model: $logit P\left( Y=1 \right|X,Z, S=1)=log\frac{P(Y=1|X,Z,S=1)}{P(Y=0|X,Z,S=1)}= \log\frac{P(Y=1|X,Z)}{P(Y=0|X,Z)}=\beta_{0}+\beta_{1}X+\beta_{2}Z$


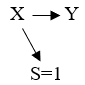


Scenario 3 A DAG representing marginally dependent X and Y, and conditionally (on S = 1) dependent X.

(4) Scenario 4：Suppose the selection of participants is affected by Y and X.

Selection model: $P(S=1|Y,X,Z)= P(S=1|Y, X,Z)$

Outcome model: $logit P\left( Y=1 \right|X,Z, S=1)=log\frac{P(Y=1|X,Z,S=1)}{P(Y=0|X,Z,S=1)}=log\frac{{P(Y=1|X,Z)P(S=1|X,Z,Y=1)}/{[P(Y=0|X,Z)P(S=1|X,Z,Y=0)+P(Y=1|X,Z)P(S=1|X,Z,Y=1)]}}{{P(Y=0|X,Z)P(S=1|X,Z,Y=0)}/{[P(Y=0|X,Z)P(S=1|X,Z,Y=0)+P(Y=1|X,Z)P(S=1|X,Z,Y=1)]}}= \beta_{0}{+ \beta}_{1}X+\beta_{2}Z+ (\alpha_{1}+log\frac{1+e^{\alpha_{0}+\alpha_{2}X}}{1+e^{\alpha_{0+}\alpha_{1}+\alpha_{2}X}})$


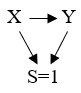


Scenario 4 A DAG representing marginally dependent X and Y, and conditionally (on S = 1) dependent X and Y.

(5) Scenario 5：Suppose the selection of participants is affected by Y, X and Z. Z is a confounder variable.

Selection model: $P(S=1|Y,X,Z)= P(S=1|Y, X,Z)$

Outcome model: $logit P\left( Y=1 \right|X,Z, S=1)=log\frac{P(Y=1|X,Z,S=1)}{P(Y=0|X,Z,S=1)}=log\frac{{P(Y=1|X,Z)P(S=1|X,Z,Y=1)}/{[P(Y=0|X,Z)P(S=1|X,Z,Y=0)+P(Y=1|X,Z)P(S=1|X,Z,Y=1)]}}{{P(Y=0|X,Z)P(S=1|X,Z,Y=0)}/{[P(Y=0|X,Z)P(S=1|X,Z,Y=0)+P(Y=1|X,Z)P(S=1|X,Z,Y=1)]}}= \beta_{0}{+ \beta}_{1}X+\beta_{2}Z+ (\alpha_{1}+log\frac{1+e^{\alpha_{0}+\alpha_{2}X+\alpha_{3}Z}}{1+e^{\alpha_{0+}\alpha_{1}+\alpha_{2}X+\alpha_{3}Z}})$


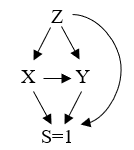


Scenario 5 A DAG representing marginally dependent X and Y with additional conditional (on S = 1) dependency and confounding variable Z directly affects S.

(6) Scenario 6：Suppose the selection of participants is affected by Y, X and Z. Z is an effect measure modifier.

Selection model: $P(S=1|Y,X,Z,XZ)= P(S=1|Y, X,Z,XZ)$

Outcome model: $logit P\left( Y=1 \right|X,Z,XZ, S=1)=log\frac{P(Y=1|X,Z,XZ,S=1)}{P(Y=0|X,Z,XZ,S=1)}=log\frac{{P(Y=1|X,Z,XZ)P(S=1|X,Z,XZ,Y=1)}/{[P(Y=0|X,Z,XZ)P(S=1|X,Z,XZ,Y=0)+P(Y=1|X,Z,XZ)P(S=1|X,Z,XZ,Y=1)]}}{{P(Y=0|X,Z,XZ)P(S=1|X,Z,XZ,Y=0)}/{[P(Y=0|X,Z,XZ)P(S=1|X,Z,XZ,Y=0)+P(Y=1|X,Z,XZ)P(S=1|X,Z,XZ,Y=1)]}}= \beta_{0}{+ \beta}_{1}X+\beta_{2}Z+\beta_{3}XZ+ (\alpha_{1}+log\frac{1+e^{\alpha_{0}+\alpha_{2}X+\alpha_{3}Z+\alpha_{4}XZ}}{1+e^{\alpha_{0+}\alpha_{1}+\alpha_{2}X+\alpha_{3}Z+\alpha_{4}XZ}})$


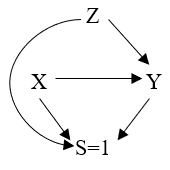


Scenario 6 A DAG representing marginally dependent X and Y with additional conditional (on S = 1) dependency, and effect measure modifier Z affects S.

The interaction between the effects of X and Z influences on both S and Y. The interaction between the effects of X and Z is also influenced by both X and Z.
